# Gene Expression Profiling of *Clostridium botulinum* under Heat Shock Stress

**DOI:** 10.1155/2013/760904

**Published:** 2013-09-30

**Authors:** Wan-dong Liang, Yun-tian Bi, Hao-yan Wang, Sheng Dong, Ke-shen Li, Jin-song Li

**Affiliations:** ^1^College of Life Science, Zhejiang Provincial Key Laboratory of Medical Genetics, Wenzhou Medical University, Wenzhou 325000, China; ^2^School of Basic Medical Science, Wenzhou Medical University, Wenzhou 325000, China; ^3^Neurosurgery, The First Hospital of Jiamusi University, Jiamusi 154007, China; ^4^Cardiovascular Surgery, The Fourth Hospital of Harbin Medical University, Harbin 157003, China; ^5^Clinical Medicine Research Center, Affiliated Hospital of Guangdong Medical College, Zhanjiang 524001, China

## Abstract

During growth, *C. botulinum* is always exposed to different environmental changes, such as temperature increase, nutrient deprivation, and pH change; however, its corresponding global transcriptional profile is uncharacterized. This study is the first description of the genome-wide gene expression profile of *C. botulinum* in response to heat shock stress. Under heat stress (temperature shift from 37°C to 45°C over a period of 15 min), 176 *C. botulinum* ATCC 3502 genes were differentially expressed. The response included overexpression of heat shock protein genes (*dnaK* operon, *groESL*, *hsp20,* and *htpG*) and downregulation of aminoacyl-tRNA synthetase genes (*valS*, *queA*, *tyrR*, and *gatAB*) and ribosomal and cell division protein genes (*ftsZ* and *ftsH*). In parallel, several transcriptional regulators (*marR*, *merR*, and *ompR* families) were induced, suggesting their involvement in reshuffling of the gene expression profile. In addition, many ABC transporters (oligopeptide transport system), energy production and conversion related genes (*glpA* and *hupL*), cell wall and membrane biogenesis related genes (*fabZ*, *fabF*, and *fabG*), flagella-associated genes (*flhA*, *flhM*, *flhJ*, *flhS*, and *motAB*), and hypothetical genes also showed changed expression patterns, indicating that they may play important roles in survival under high temperatures.

## 1. Introduction

*Clostridium botulinum* is a Gram-positive, rod-shaped bacterium that can produce neurotoxins, which cause the flaccid muscular paralysis seen in botulism [1]. As a neuroparalytic disease, botulism occurs following ingestion of food contaminated by *C*. *botulinum* neurotoxin or by other means of toxin exposure [2]. In addition, *C. botulinum* can colonize wounds and the adult/infant gastrointestinal tract and produce neurotoxins, leading to wound botulism and adult/infant botulism, respectively. 

Upon encountering a sudden but tolerable temperature increase, bacteria can survive and adapt to the new temperature through differential expression of their encoded genes, leading to the heat shock response [3, 4]. For example, about 15% of the total *Shewanella oneidensis* genes were shown to be significantly differentially expressed in response to a temperature increase from 30°C to 42°C over a period of 25 min, including chaperones, enzymes of glycolysis, transcriptional regulators, and histidine kinases [5]. Global transcriptional profile analysis of *Francisella tularensis* revealed that about 11% of its total genes were differentially regulated, including heat shock proteins, transcriptional regulators, and virulence-associated genes [6]. For *C*. *botulinum,* previous studies indicated that the expression of molecular chaperones, such as *dnaJ, groEL,* and *groES,* and protein synthesis were altered upon temperature increase from 37°C to 45°C to enable cells to survive at high temperature [7–10]. The results suggested that differential gene expression was critical to *C*. *botulinum* to allow it to grow in environments with high temperatures. However, the global transcriptional profile of *C. botulinum* upon temperature increase has not been determined. 

In this study, we determined the global transcriptional profile of *C. botulinum* under high temperature stress using expression microarray analysis to enhance our understanding of the capacity of *C. botulinum* to adapt to high temperatures and to identify candidate genes involved in the heat shock response for further functional characterization. 

## 2. Materials and Methods

### 2.1. Bacterial Strain and Growth Conditions

*C. botulinum* ATCC 3502 was grown anaerobically at 37°C in trypticase-peptone-glucose-yeast extract (TPGY) medium supplemented with 2.5 *μ*g/mL erythromycin, 250 *μ*g/mL cycloserine, or 15 *μ*g/mL thiamphenicol (all antibiotics from Sigma-Aldrich, Steinheim, Germany) in an anaerobic chamber (Bugbox; Ruskinn Technology, Bridgend, UK) [9]. For the heat shock treatment, a single colony was inoculated into the anaerobic TPGY medium and incubated at 37°C until midexponential growth phase (corresponding to an optical density at 600 nm (OD_600_) of 0.98). Aliquots of the cultures were then heat shocked at 45°C in a water bath for 15 min under anaerobic conditions. 

### 2.2. RNA Extraction, cDNA Synthesis, and Hybridization

Total RNA was extracted separately from each culture using TRIzol reagent (Invitrogen) following the manufacturer's instructions. Extracted RNA was treated with DNase I (Ambion, Austin, TX, USA) and gel purified (RNeasy Mini kit, QIAGEN). The quality of the RNA samples was measured spectrophotometrically. A double-stranded cDNA library, which was used for the expression analysis, was produced by reverse transcription from 10 *μ*g of total RNA using the SuperScript Double-Stranded cDNA Synthesis Kit (Invitrogen). Double-stranded cDNA was cleaned and labeled using the NimbleGen genes expression analysis protocol (NimbleGen Systems, Madison, WI, USA). 

Expression microarray analysis was performed using a custom-made NimbleGen array, designed based on the genomic sequence of *C. botulinum* ATCC 3502 [11]. It contained 18 replicates of each 60-mer oligonucleotide probe designed for the 3,669 genes. The whole genome was represented five times on each chip (five technical replicates), which approximates to 90 probes per genes. For hybridization, 5 *μ*g of double-stranded cDNA was hybridized with NimbleGen hybridization buffer and NimbleGen hybridization component A in hybridization chambers (TeleChem International, Sunnyvale, CA, USA) overnight at 42°C. Washing was then performed using NimbleGen wash solutions I, II, and III, according to the manufacturer's protocol.

### 2.3. Microarray Data Analysis

An Axon GenePix 4000B scanner (Molecular Devices Corporation, Sunnyvale, CA, USA) and NimbleScan program v2.5 (Madison, WI, USA) were used for image scanning and subsequent generation of raw expression data. Three biological replicates and five technical replicates were investigated for each transcriptional condition, resulting in eight measurements for each genes. The expression data for each transcriptional condition were the average values of all nine measurements. LNN, a flexible empirical Bayes model in the EB arrays package (R/Bioconductor, http://www.bioconductor.org/), was used for the statistical analysis. For a genes to be classified as differentially expressed, *P* values had to be less than 0.0001, and the fold-difference in hybridization signal intensities had to be greater than two.

### 2.4. Quantitative Real-Time PCR

To confirm the differentially expressed genes obtained from the microarray expression profile, 15 genes were analyzed by quantitative real-time PCR (qRT-PCR) using the Bio-Rad CFX96 system. *gyrB* (CBO0006) was used as the endogenous control. The 15 selected genes included eight upregulated genes (CBO2958, CBO1012, CBO0787, CBO0831, CBO1677, CBO3570, CBO0377, and CBO0043) and seven downregulated genes (CBO1840, CBO3520, CBO3465, CBO2101, CBO0400, CBO2645, and CBO3069). The corresponding primers (see Table S1 in supplementary Material available online at http://dx.doi.org/10.1155/2013/760904) were designed using Primer Premier software version 5 (PREMIER Biosoft International, Palo Alto, CA, USA). Two micrograms of total RNA were used as a template for cDNA synthesis with Superscript III (Invitrogen). The amplification programs were performed as recommended by the standard protocol of the Bio-Rad CFX96 system: 98°C for 2 min; 98°C for 2 s, 59°C for 10 s, for 40 cycles; followed by a thermal denaturing step to generate the melt curves for verification of amplification specificity. All reactions were performed in triplicate, and statistical analysis was performed using the Ct (2^−ΔΔCt^) method [12].

## 3. Results and Discussion

### 3.1. Differential Expression of *C. botulinum* Genes under Heat Shock Stress

*C. botulinum* is a pathogenic bacterium that produces a life-threatening toxin causing botulism [1, 2]. To investigate genome-wide differential genes expression under heat shock stress, a microarray was used to study expression levels and identify differentially expressed genes. *C. botulinum* ATCC 3502 was collected and used for microarray hybridization and expression analysis by shifting the temperature from 37°C to 45°C over a period of 15 min. In comparison with those expressed at 37°C, 176 genes were differentially expressed at 45°C with more than two-fold differences in expression level and a *P* value <0.0001, including 84 upregulated genes and 92 downregulated genes (Table S2). 

**Figure 1:**
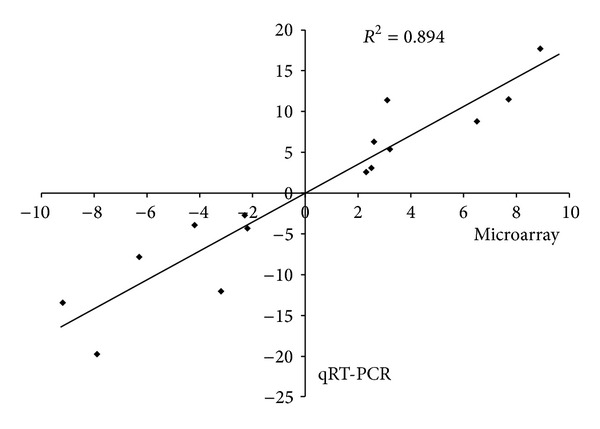
Correlations of the differential expression ratios between microarray and qRT-PCR.

To validate the reliability of the expression level obtained from the microarray analysis, 15 differentially expressed genes (eight upregulated and seven downregulated) were randomly chosen for qRT-PCR analysis. All of these genes exhibited the same pattern of differential expression in qRT-PCR and microarray. As shown in Figure 1, a strong correlation (Pearson's correlation 0.894) was found between the microarray analysis and the qRT-PCR results, which indicated a good concordance of both methods. However, it should be noted that the quantitative-fold differences between the methods were different and the genes expression level based on the microarray analysis was relatively low compared with that of qRT-PCR. Underestimation of fold changes by microarray analysis has been reported previously, reflecting the higher sensitivity of qRT-PCR [5, 13].

### 3.2. Functional Classification of Differentially Expressed Genes

**Figure 2:**
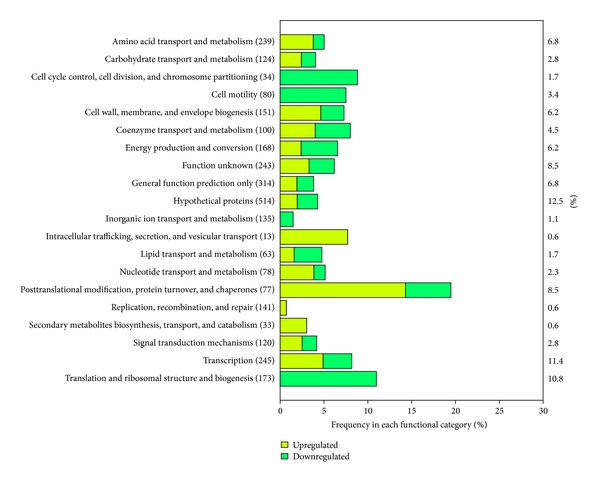
The COG functional categorization of differentially expressed genes in response to heat shock stress in *Clostridium botulinum* ATCC 3502. The number in parentheses showed the total number of genes classified in each COG functional category in *Clostridium botulinum* ATCC 3502. The bar diagram represented the percentage of differentially expressed genes (including upregulated and down-regulated genes) relative to the total number of genes in each COG functional category. The number beside the bar diagram indicated the percentage of differentially expressed genes in each COG functional category relative to the total number of differentially expressed genes under heat shock stress.

To obtain an overall insight into the impact of heat shock stress on *C. botulinum* ATCC 3502, the 176 differentially expressed genes were functionally annotated based on COG (Clusters of Orthologous Groups, http://www.ncbi.nlm.nih.gov/COG/) classification. Among them, 154 differentially expressed genes were assigned a function based on COG classification, which may represent important stress-related functions required for the survival of *C. botulinum* in high temperature. For genes with annotated functions, the most upregulated were those encoding proteins involved in transcription and posttranslational modification, protein turnover, and chaperones (Figure 2). In contrast, genes encoding proteins involved in transcription and translation and ribosomal structure and biogenesis were among the most downregulated genes. In addition, many differentially expressed genes were classified as hypothetical function, function unknown, and general function prediction only, consistent with the high proportion of uncharacterized genes in *C. botulinum* ATCC 3502. The 50 most significant differentially expressed genes under temperature change are listed in Table 1.

### 3.3. Posttranslational Modification, Protein Turnover, and Chaperone Genes

Searching the whole genome of *C. botulinum* ATCC 3502 revealed 81 genes for chaperones or proteins involved in posttranslational modification or protein turnover, representing 2.85% of the total gene number [11]. Similar to previous work [5, 6], many chaperones were differentially expressed in response to heat shock in *C. botulinum* ATCC 3502, including most of the genes belonging to the class I heat shock proteins. Of the six chaperone genes with upregulated transcript levels, *dnaJ* (CBO2958), *dnaK* (CBO2959), DnaJ-related protein (CBO0189), and *grpE* (CBO2960) showed more than 5-fold induction, while their transcriptional regulator *hrcA* (CBO2961) was 3.5-fold upregulated under heat stress. According to the genes organization from MicrobesOnline [14], these genes comprise an operon and have an upstream CIRCE inverted repeat (TTAGCACTC-N_9_-GAGTGCTAA) that is essential for HrcA binding. The importance of overexpressing DnaK and HrcA for survival in a high temperature environment has recently been validated in *C. botulinum* ATCC 3502, where constitutive overexpression of the *dnaK* operon significantly enhanced resistance to high temperature [9]. In addition, two chaperone protein genes, *groEL* (CBO3298) and *groES* (CBO3299), were upregulated by 8.3-fold and 14.2-fold, respectively, upon temperature change [7]. According to the genes organization, both genes also form an operon and have an upstream CIRCE inverted repeat (TTAGCACTC-N_9_-GAGTGCTAA) that is essential for HrcA binding. These observations suggested that overexpression of class I heat shock genes plays an important role in the ability of *C. botulinum* ATCC 3502 to survive and grow under heat stress conditions. 

In addition to class I heat shock genes, other heat shock-related genes, such as *hsp20* (CBO0831) and *htpG* (CBO1985), were also significantly upregulated in *C. botulinum* ATCC 3502 in response to temperature increase. These heat shock genes are associated with protein folding, such as degrading improperly folded proteins, and cell partitioning, such as associating with DNA structure [15]. A previous study indicated that the subunits of the Clp protease complex were often differentially expressed during heat shock stress [16]. However, in this study, none of the genes encoding the subunits of the Clp protease complex, such as *clpB, clpX, clpP,* and *clpC,* were differentially expressed upon heat shock. 

In addition, previous studies showed that *C. botulinum* ATCC 3502 alters the protein expression of botulinum toxin cluster genes *ha-33* under temperature shift from 37°C to 45°C, indicating that *ha-33* is a heat shock protein [8, 10]. In this study, we found that none of the toxin cluster genes was identified to be differentially expressed based on the 2-fold difference in expression level and *P*  value less than 0.0001 criteria. However, two toxin cluster genes, *ha-17* (CBO0802) and *ha-33* (CBO0803), were upregulated by 1.6- and 1.8-fold, respectively, under heat stress in the microarray study. Although both of them were under the cutoff value of 2-fold, this suggests that they may also play important roles through protecting and stabilizing neurotoxins under high temperature conditions [10]. 

### 3.4. Translation, Ribosomal Structure, and Biogenesis Genes

Several of the significantly overexpressed genes were assigned to COG functional category J (translation, ribosomal structure, and biogenesis). This category mainly includes genes involved in the translation machinery, such as aminoacyl-tRNA synthetase and ribosomal proteins. We found that many genes involved in the translation machinery were downregulated, indicating that heat shock stress slowed translation and reduced energy needs and cellular physiology in *C. botulinum* ATCC 3502. Among them, *valS* (CBO3164, valyl-tRNA synthetase), *queA* (CBO3069, S-adenosylmethionine), and *tyrR* (CBO1204, tyrosyl-tRNA synthetase) were the three most significantly downregulated genes (by 12.1-, 9.2-, and 5.6-fold, resp.). In addition, *gatB* (CBO3265, aspartyl/glutamyl-tRNA amidotransferase subunit B1), *gatA* (CBO3266, glutamyl-tRNA amidotransferase subunit A), and *gatC* (CBO3267, glutamyl-tRNA amidotransferase subunit C) compose an operon and were downregulated by heat shock stress. Moreover, *proS2* (CBO3503, prolyl-tRNA synthetase), *aspS* (CBO1019, aspartyl-tRNA synthetase), and *tyrS* (CBO3323, tyrosyl-tRNA synthetase) were also downregulated upon a temperature increase from 37°C to 45°C.

Several genes involved in ribosomal protein synthesis and modification were downregulated, including *rpmE* (CBO0135), *rplR* (CBO3465), *rplO* (CBO3462), *rplX* (CBO3470), *rpsQ* (CBO3472), *rpsN* (CBO3468), *rpsC* (CBO3474), and *rplK* (CBO3492). The downregulation of ribosomal genes indicated a temporary growth arrest, allowing the bacteria to reshuffle energy to adapt to the higher temperature [13]. Furthermore, this decrease in ribosomal proteins may result in a modest repression of *de novo* protein synthesis, which would allow the bacteria to overcome heat shock stress and adapt to the higher temperature growth conditions. Consistent with this hypothesis, several genes involved in cell cycle control, cell division, and chromosome partitioning (COG category D) were rapidly downregulated. Among them, *ftsZ* (CBO2535), encoding a protein that is pivotal for cell division in many bacteria [17], was downregulated by 4.7-fold upon temperature increase. In addition, ftsH (CBO0400), encoding an ATP- and Zn^2+^-dependent metalloprotease that is essential for viability in *Escherichia coli* [18], was downregulated by 6.3-fold. 

### 3.5. Transcriptional Regulators

Several genes encoding transcriptional regulators were differentially expressed in response to heat shock stress, suggesting that they have essential roles under heat shock stress in *C. botulinum* ATCC 3502. Searching the annotation of all coding genes in *C. botulinum* ATCC 3502, 13 genes belonging to the MarR family [11], which serve as transcriptional repressors, were identified. Under heat shock stress, three of these genes (CBO0645, CBO1012, and CBO1016) were differentially expressed. The expression levels of CBO0645 and CBO1012 were elevated by 16.1- and 7.7-fold, respectively, upon temperature shift. However, CBO1016 was downregulated under heat shock stress, indicating a distinct function of the MarR family in the regulation of heat shock in *C. botulinum* ATCC 3502. A previous study indicated that *marR* served as a repressor for the *marRAB* operon in *E. coli* and regulated resistance to multiple antibiotics in this organism [19]. Inactivation of *marR* can lead to increased expression of *marA,* which binds to many target genes and reduces antibiotic accumulation [19]. Interestingly, adjacent to CBO1016 is another transcriptional regulator, CBO1017, belonging to the MerR family. CBO1017 was decreased about 5.8-fold in response to heat shock stress. According to the predicted operon architecture from MicrobesOnline [14], the genes are organized into an operon and cotranscribed. However, the functional roles of the MarR and MerR families are uncharacterized in *C. botulinum* ATCC 3502; therefore, they will provide a basis for future in-depth functional studies. CBO2304 and CBO2164, predicted to belong to the transcriptional regulator PadR and AraC family, were also differentially expressed. 

The two-component signal transduction system, consisting of a sensor kinase and a response regulator, represents a major paradigm for signal transduction across many bacteria [20]. In these systems, the response regulator often acts as a transcriptional regulator. Under heat shock stress, two response regulators, CBO0786 and CBO3308, both belonging to the transcriptional regulator OmpR family, were overexpressed. Meanwhile, their corresponding adjacent kinases (CBO0787 and CBO3309, respectively) were also overexpressed under high temperature stress. This observation indicated that two-component signal transduction has important roles in the adaptation of *C. botulinum* ATCC 3502 to heat shock stress. In addition, transcriptional regulators *ccpA* (CBO0100, LacI family) and *treR* (CBO1989, GntR family), which control the efficiency of glucose and trehalose metabolism in *Clostridium difficile* [21], respectively, were downregulated.

In addition, *sigH* (CBO3497) and *rpoE* (CBO0043) (encoding the RNA polymerase sigma H and sigma E proteins, resp.) were 3.5- and 2.3-fold upregulated, respectively. Sigma factors are directly involved in the transcription of specific sets of genes, and their expression is induced in response to stress. In *E. coli*, *rpoE* was revealed to be important for its growth at high temperatures [22], while in *Salmonella enterica* Serovar Typhimurium, *rpoE* was related to survival of the bacterium under nutritional deprivation and oxidative stress, where it was strongly expressed upon reaching stationary phase [23]. Moreover, the major sigma factor, *rpoD* (CBO2938), was upregulated.

### 3.6. Hypothetical Proteins

The genes annotation of the *C. botulinum* ATCC 3502 genome shows a large percentage (34.5%) of coding genes with no functional annotation [11]. In this study, differentially expressed genes for hypothetical proteins accounted for 21.4% and 20.7% of upregulated and downregulated genes, respectively. The majority of these genes were from poorly characterized single-genes operons or polypeptides. For other operons containing multiple genes encoding hypothetical proteins, we observed consistent coregulation of several members of the operons, such as CBO1070-*glpT*, *greA*-CBO3520-CBO3521-CBO3522, and CBO3194-CBO3195. The protein encoded by *glpT* (CBO1071, glycerol-3-phosphate transporter), which transports glycerol-3-phosphate into the cytoplasm and inorganic phosphate into the periplasm, was upregulated. The other member of this operon (CBO1070) was also highly induced under heat shock stress. Therefore, it is likely that the function of hypothetical protein CBO1070 may be associated with the transporter. *greA* (CBO3519) is a transcription elongation factor that stimulates elongation of RNA [24], whereas CBO3521 encodes a dihydrouridine synthase, and CBO3520 and CBO3522 are hypothetical proteins. All the members of this operon were coregulated. Therefore, future studies are needed to reveal the roles of these proteins and how these genes are controlled under heat shock stress. 

### 3.7. Miscellaneous Observations

Several genes related to various kinds of metabolic pathways were differentially expressed under high temperature in *C. botulinum* ATCC 3502. For example, *glpA* (CBO1068; glycerol-3-phosphate dehydrogenase) and *glpT* (CBO1071; a transporter in glycerol metabolism) were upregulated at 45°C. *ptsH* (CBO2398) and *ptsI* (CBO3438), two genes in the phosphotransferase system, were also upregulated. The upregulation of genes encoding proteins involved in metabolism reflected the increased expression of proteins involved in damage repair to compensate for the decrease in activity and stability caused by heat stress [25].

ABC transporters were also differentially expressed under heat stress. In particular, all members of an operon comprising CBO1675 (oligopeptide-binding protein), CBO1676 (oligopeptide transport system, permease protein), CBO1677 (oligopeptide transport system, permease protein), and CBO1678 (oligopeptide transport system, ATP-binding protein) were upregulated. This suggested that the oligonucleotide transport system may have important functions in providing essential amino acids for the growth of *C. botulinum* ATCC 3502 at high temperatures. Meanwhile, *ecsC *(CBO1860) and CBO3174, encoding the membrane protein responsible for inserting integral membrane proteins into the membrane, were upregulated during heat stress. Their upregulation may reflect the need to increase protein translocation in *C. botulinum* ATCC 3502, either to stabilize or to maintain the integrity of membrane proteins.

The expression of five genes involved in energy production and conversion, CBO1840 (*hupL,* hydrogenase large subunit), CBO3183 (pyruvate formate-lyase), CBO3184 (pyruvate formate-lyase 2 activating enzyme), CBO3577 (nitroreductase), CBO0155 (*atpG,* ATP synthase subunit gamma), and CBO0207 (iron-sulfur cluster-binding protein), was downregulated. This suggests that a reduction in energy-requiring processes in *C. botulinum* ATCC 3502 may represent a survival strategy of bacteria during heat shock stress [26]. 

Several genes involved in cell wall and membrane biogenesis were differentially expressed, indicating that formation and remodeling of the cell wall were essentially to prevent cell lysis during heat shock in *C. botulinum.* For example, CBO1424 (N-acetylmuramoyl-L-alanine amidase), CBO2101 (UDP-glucose epimerase), and CBO1454 (S-adenosyl-methyltransferase) were downregulated by 2- to 5-fold following heat shock. CBO3562 (glutamate racemase), CBO1660 (alanine racemase), and CBO0380 (cell surface protein) were all upregulated. One of the most highly overexpressed genes (increased by 8.3-fold) was *fabZ* (CBO3597), encoding the (3R)-hydroxymyristoyl-ACP dehydratase, which is associated with fatty acid biosynthesis. In *E. coli*, *fabZ* is part of the sigma E regulon and is also induced under heat shock [27]. In addition, two genes belonging to the same operon, *fabF* (CBO3599, 3-oxoacyl-ACP synthase II) and *fabG* (CBO3600, 3-oxoacyl-ACP reductase), were upregulated by 4.2- and 2.3-fold, respectively. 

Flagella are the most widely characterized of the bacterial motility structures [28]. Interestingly, we found that four genes involved in flagella biosynthesis were differentially expressed. For example, *flhA* (CBO2645), encoding a protein that is part of the export apparatus for flagellum assembly, was among the most highly differentially expressed genes involved in flagella. In addition, *motB-motA* (CBO2651-CBO2652) and *fliM* (CBO2744), involved in the motor function of flagella, were downregulated by 2.3- to 3.6-fold under heat shock stress. Meanwhile, the general components of the flagellar export apparatus, *fliJ* (CBO2658) and *fliS* (CBO2734), which are related to the chaperone for rod and hook proteins, were also differentially expressed. The nature of the differential expression of these flagellar genes in the heat shock response remains unknown.

Several genes encoding proteins involved in antioxidant function were upregulated during heat stress. Among them, *tpx* (CBO0501), a thiol peroxidase, was the most significantly induced, which was consistent with a previous report that heat shock stress causes an antioxidant response [29].

## Supplementary Material

Table S1. The primers for quantitative real-time PCR
Table S2. Genes induced two folds or more different in response to heat shock stress in Clostridium botulinum ATCC 3502

## Figures and Tables

**Table 1:** The top 40 differentially expressed genes in response to heat shock stress in *Clostridium botulinum* ATCC 3502.

Locus tag	COG	Gene name	Function class	Fold change	Function description
Posttranslational modification, protein turnover, and chaperones [O]					
CBO2958	COG0484	*dnaJ *	[O]	+8.9	Chaperone protein
CBO2959	COG0443	*dnaK *	[O]	+17.2	Chaperone protein
CBO0189	COG0484		[O]	+8.2	DnaJ-related protein
CBO2960	COG0576	*grpE *	[O]	+5.6	Heat shock protein
CBO3299	COG0234	*groES *	[O]	+14.2	10 kDa chaperonin GroES
CBO3298	COG0459	*groEL *	[O]	+8.3	60 kDa chaperonin GroEL
CBO0501	COG2077	*tpx *	[O]	+5.0	Thiol peroxidase
CBO0803		*ha-33 *		+1.8	Toxin cluster gene *ha-33 *
CBO0802		*ha-17 *		+1.6	Toxin cluster gene *ha-17 *

Transcription [K]					
CBO0645	COG1846		[K]	+16.1	MarR-family transcriptional regulator
CBO1012	COG1846		[K]	+7.7	MarR-family transcriptional regulator
CBO1017	COG4978		[KT]	−5.8	MerR-family transcriptional regulator
CBO2304	COG1695		[K]	+5.8	Transcriptional regulator, PadR family
CBO0786	COG0745		[KT]	+4.4	Two component response regulator
CBO0100	COG1609	*ccpA *	[K]	−7.3	LacI-family transcriptional regulator
CBO2938	COG0568	*rpoD *	[K]	+4.2	RNA polymerase sigma factor (sigma-43)
CBO3519	COG0782	*greA *	[K]	−5.0	Transcription elongation factor

Translation and ribosomal structure and biogenesis [J]					
CBO3164	COG0525	*valS *	[J]	−12.1	Valyl-tRNA synthetase
CBO3069	COG0809	*queA *	[J]	−9.2	tRNA ribosyltransferase-isomerase
CBO1204	COG0162	*tyrR *	[J]	−5.6	Tyrosyl-tRNA synthetase
CBO3265	COG0064	*gatB *	[J]	−4.3	Glutamyl-tRNA amidotransferase subunit B
CBO3503	COG0442	*proS2 *	[J]	−4.3	Prolyl-tRNA synthetase
CBO3323	COG0162	*tyrS *	[J]	−4.7	Tyrosyl-tRNA synthetase
CBO3470	COG0198	*rplX *	[J]	−6.7	50S ribosomal protein L24
CBO3474	COG0092	*rpsC *	[J]	−4.6	30S ribosomal protein S3
CBO0400	COG0465	*ftsH *	[J]	−6.3	Cell division protein

Amino acid transport and metabolism [E]					
CBO1675	COG0747		[E]	+6.8	Oligopeptide-binding protein
CBO1860	COG0697	*ecsC *	[E]	+4.1	Membrane protein

Cell wall, membrane, and envelope biogenesis [M]					
CBO2101	COG1087		[M]	−4.2	UDP-glucose epimerase
CBO3597	COG0764	*fabZ *	[M]	+8.3	(3R)-Hydroxymyristoyl-ACP dehydratase

Hypothetical proteins					
CBO3522				−4.1	Hypothetical protein
CBO2185				+5.3	Hypothetical protein
CBO2640				−6.3	Hypothetical protein
CBO2096				+5.8	Hypothetical protein
CBO3353				+7.6	Hypothetical protein

Function unknown [S]					
CBO1070	COG3862		[S]	+6.0	Hypothetical protein
CBO0014	COG3862		[S]	−4.2	Hypothetical protein
CBO2935	COG0327		[S]	+4.1	Hypothetical protein

General function prediction only [R]					
CBO1449	COG2234		[R]	+4.6	Membrane protein
CBO0670	COG2984		[R]	−4.2	Lipoprotein
CBO2137	COG2234		[R]	+4.7	Membrane protein

Cell motility [N]					
CBO2645	COG1298	*flhA *	[NU]	−7.9	Flagellar biosynthesis protein
CBO2652	COG1291	*motA *	[N]	−5.2	Chemotaxis MotA protein

Energy production and conversion [C]					
CBO3577	COG0778		[C]	−5.2	Nitroreductase

Signal transduction mechanisms [T]					
CBO0787	COG0642		[T]	+6.5	Two-component sensor kinase
CBO1120	COG0642		[T]	+4.3	Two-component sensor kinase

Cell cycle control, cell division, and chromosome partitioning [D]					
CBO2535	COG0206	*ftsZ *	[D]	−4.7	Cell division protein FtsZ

Coenzyme transport and metabolism [H]					
CBO2911	COG0422	*thiC *	[H]	−4.3	Thiamine biosynthesis protein
CBO2601	COG1057		[H]	+4.6	Transferase

Nucleotide transport and metabolism [F]					
CBO0132	COG0504	*pyrG *	[F]	−4.7	CTP synthase

Carbohydrate transport and metabolism [G]					
CBO1071	COG2271	*glpT *	[G]	+4.3	Glycerol-3-phosphate transporter

Secondary metabolites biosynthesis, transport, and catabolism [Q]					
CBO1169	COG2313		[Q]	+4.3	Indigoidine synthase A family protein

## References

[1] Fang P-K, Raphael BH, Maslanka SE, Cai S, Singh BR (2010). Analysis of genomic differences among Clostridium botulinum type A1 strains. *BMC Genomics*.

[2] Lindström M, Kiviniemi K, Korkeala H (2006). Hazard and control of group II (non-proteolytic) Clostridium botulinum in modern food processing. *International Journal of Food Microbiology*.

[3] Ferenci T, Spira B (2007). Variation in stress responses within a bacterial species and the indirect costs of stress resistance. *Annals of the New York Academy of Sciences*.

[4] Rangel DEN (2011). Stress induced cross-protection against environmental challenges on prokaryotic and eukaryotic microbes. *World Journal of Microbiology and Biotechnology*.

[5] Gao H, Wang Y, Liu X (2004). Global transcriptome analysis of the heat shock response of Shewanella oneidensis. *Journal of Bacteriology*.

[6] Horzempa J, Carlson PE, O’Dee DM, Shanks RMQ, Nau GJ (2008). Global transcriptional response to mammalian temperature provides new insight into Francisella tularensis pathogenesis. *BMC Microbiology*.

[7] Sagane Y, Hasegawa K, Mutoh S (2003). Molecular characterization of GroES and GroEL homologues from Clostridium botulinum. *Journal of Protein Chemistry*.

[8] Shukla HD, Singh BR (1999). Identification of DnaJ-like chaperone in Clostridium botulinum type A. *Journal of Protein Chemistry*.

[9] Selby K, Lindström M, Somervuo P, Heap JT, Minton NP, Korkeala H (2011). Important role of class I heat shock genes hrcA and dnaK in the heat shock response and the response to pH and NaCl stress of group I Clostridiusm botulinum strain ATCC 3502. *Applied and Environmental Microbiology*.

[10] Shukla HD, Singh BR (2009). Hemagglutinin-33 in the neurotoxin complex of typec A Clostridium botulinum is a Heat Shock Protein. *Botulinum Journal*.

[11] Sebaihia M, Peck MW, Minton NP (2007). Genome sequence of a proteolytic (Group I) Clostridium botulinum strain Hall A and comparative analysis of the clostridial genomes. *Genome Research*.

[12] Livak KJ, Schmittgen TD (2001). Analysis of relative gene expression data using real-time quantitative PCR and the 2-ΔΔCT method. *Methods*.

[13] Stintzi A (2003). Gene expression profile of Campylobacter jejuni in response to growth temperature variation. *Journal of Bacteriology*.

[14] Dehal PS, Joachimiak MP, Price MN (2009). MicrobesOnline: an integrated portal for comparative and functional genomics. *Nucleic Acids Research*.

[15] Fink AL (1999). Chaperone-mediated protein folding. *Physiological Reviews*.

[16] Frees D, Savijoki K, Varmanen P, Ingmer H (2007). Clp ATPases and ClpP proteolytic complexes regulate vital biological processes in low GC, Gram-positive bacteria. *Molecular Microbiology*.

[17] Rothfield L, Justice S, García-Lara J (1999). Bacterial cell division. *Annual Review of Genetics*.

[18] Tomoyasu T, Yuki T, Morimura S (1993). The Escherichia coli FtsH protein is a prokaryotic member of a protein family of putative ATPases involved in membrane functions, cell cycle control, and gene expression. *Journal of Bacteriology*.

[19] Sulavik MC, Gambino LF, Miller PF (1995). The MarR repressor of the multiple antibiotic resistance (mar) operon in Escherichia coli: prototypic member of a family of bacterial regulatory proteins involved in sensing phenolic compounds. *Molecular Medicine*.

[20] Stock AM, Robinson VL, Goudreau PN (2000). Two-component signal transduction. *Annual Review of Biochemistry*.

[21] Antunes A, Camiade E, Monot M (2012). Global transcriptional control by glucose and carbon regulator CcpA in Clostridium difficile. *Nucleic Acids Research*.

[22] Hiratsu K, Amemura M, Nashimoto H, Shinagawa H, Makino K (1995). The rpoE gene of *Escherichia coli*, which encodes *σ*(E), is essential for bacterial growth at high temperature. *Journal of Bacteriology*.

[23] Testerman TL, Vazquez-Torres A, Xu Y, Jones-Carson J, Libby SJ, Fang FC (2002). The alternative sigma factor *σ*E controls antioxidant defences required for Salmonella virulence and stationary-phase survival. *Molecular Microbiology*.

[24] Hsu LM, Vo NV, Chamberlin MJ (1995). Escherichia coli transcript cleavage factors GreA and GreB stimulate promoter escape and gene expression in vivo and in vitro. *Proceedings of the National Academy of Sciences of the United States of America*.

[25] Rezzonico E, Lariani S, Barretto C (2007). Global transcriptome analysis of the heat shock response of Bifidobacterium longum. *FEMS Microbiology Letters*.

[26] Han Y-H, Liu W-Z, Shi Y-Z, Lu L-Q, Xiao S-D, Zhang Q-H (2009). Gene expression profile of helicobacter pylori in response to growth temperature variation. *Journal of Microbiology*.

[27] Rhodius VA, Suh WC, Nonaka G, West J, Gross CA (2006). Conserved and variable functions of the sigmaE stress response in related genomes. *PLoS biology*.

[28] Snyder LAS, Loman NJ, Fütterer K, Pallen MJ (2009). Bacterial flagellar diversity and evolution: seek simplicity and distrust it?. *Trends in Microbiology*.

[29] Abrashev RI, Pashova SB, Stefanova LN, Vassilev SV, Dolashka-Angelova PA, Angelova MB (2008). Heat-shock-induced oxidative stress and antioxidant response in Aspergillus niger 26. *Canadian Journal of Microbiology*.

