# Supplementary material for: Gene Expression Profiling of Clostridium botulinum under Heat Shock Stress
Source: Biomed Res Int. 2013 Sep 30;2013:760904. doi: 10.1155/2013/760904 (PMC3806222; doi:10.1155/2013/760904)
Supplement: Supplementary file 1 — Table S1. The primers for quantitative real-time PCR Table S2. Genes induced two folds or more different in response to heat shock stress in Clostridium botulinum ATCC 3502 [file 760904.f1.zip › 760904/2013-09-03/Table_S1_2738_608594.doc]

| **Primer name** | **Primer sequence** |
| --- | --- |
| CBO2958-F | cttcctgcgacaaatgtggt |
| CBO2958-R | cgacacctaaagcagcttga |
| CBO1012-F | ggggaatatgaaaagtatggtg |
| CBO1012-R | gaaactgactacaatttcttgttcttc |
| CBO0787-F | tgatatggattggacagcagaag |
| CBO0787-R | cgttccttctcccatttcactt |
| CBO0831-F | cggatcttccaggaacaaaa |
| CBO0831-R | aaaaccccattattaaatgaagca |
| CBO1677-F | gctagtattaccgcagcttcc |
| CBO1677-R | tccccagcttttagatactgga |
| CBO3570-F | atagaagtggccgatgaaaaa |
| CBO3570-R | gctgatgtgggaaggtcatt |
| CBO0377-F | aggcgtccaaaatcccttaga |
| CBO0377-R | agcctctacaccctttaagct |
| CBO0043-F | actttaaacattggattgctgct |
| CBO0043-R | aaatttacgtccccttgaaagc |
| CBO1840-F | aagctatgtaaaaccgggagt |
| CBO1840-R | cctccggtatattctcctgca |
| CBO3520-F | ttgtgttagcgccaaaaactt |
| CBO3520-R | aaaagaggcttttgtatctttgg |
| CBO3465-F | tcggaacagctgaacgtcc |
| CBO3465-R | cgtgatatatgaatccgcctctg |
| CBO2101-F | agttagtggtggtgcaggat |
| CBO2101-R | acatctatctgcgctgctaga |
| CBO0400-F | tcttacggtgctagaatgcct |
| CBO0400-R | cttcagaaccgccagctttc |
| CBO2645-F | gggcttcctcatttggcttt |
| CBO2645-R | ttgaagaagatcccctcccg |
| CBO3069-F | ggagcaaaagaagaaaccggt |
| CBO3069-R | aggtagcggcatttgtccta |
| CBO0801-F | agaagaggggatggatggat |
| CBO0801-R | tttgagggagcggttttact |
| CBO0802-F | tgcaaataatcaaaaatggaatg |
| CBO0802-R | tcataaatgtcccaggcataatc |
| CBO0803-F | tcgcaatcaaaaatggacaa |
| CBO0803-R | gtttggccgccatataaatc |
| CBO0804-F | tgatttgagcaatttcaatacaca |
| CBO0804-R | ctccttaatatcttcaacaaatttca |
| CBO0805-F | gcagaaaatggaatgggaac |
| CBO0805-R | ttaggatcaatgcctccaga |

**Table S1. The primers for quantitative real-time PCR**
