# Supplementary material for: Gene Expression Profiling of Clostridium botulinum under Heat Shock Stress
Source: Biomed Res Int. 2013 Sep 30;2013:760904. doi: 10.1155/2013/760904 (PMC3806222; doi:10.1155/2013/760904)
Supplement: Supplementary file 1 — Table S1. The primers for quantitative real-time PCR Table S2. Genes induced two folds or more different in response to heat shock stress in Clostridium botulinum ATCC 3502 [file 760904.f1.zip › 760904/2013-09-03/Table_S2_2738_608595.doc]

**Table S1. Genes induced two folds or more different in response to heat shock stress in *Clostridium botulinum* ATCC 3502**

| **Locus tag** | **COG** | **Gene name** | **Function class** | **Fold change** | **Function description** |
| --- | --- | --- | --- | --- | --- |
| **Posttranslational modification, protein turnover, chaperones [O]** | | | | | |
| CBO2958 | COG0484 | dnaJ | [O] | +8.9 | chaperone protein |
| CBO2959 | COG0443 | dnaK | [O] | +17.2 | chaperone protein |
| CBO0189 | COG0484 |  | [O] | +8.2 | DnaJ-related protein |
| CBO2960 | COG0576 | grpE | [O] | +5.6 | heat shock protein |
| CBO3299 | COG0234 | groES | [O] | +14.2 | 10 kDa chaperonin GroES |
| CBO3298 | COG0459 | groEL | [O] | +8.3 | 60 kDa chaperonin GroEL |
| CBO0831 | COG0071 | hsp20 | [O] | +3.2 | heat shock protein |
| CBO1985 | COG0326 | htpG | [O] | +2.8 | chaperone protein |
| CBO0501 | COG2077 | tpx | [O] | +5.0 | thiol peroxidase |
| CBO1833 | COG0309 | hypE | [O] | -2.1 | hydrogenase isoenzymes formation protein |
| CBO1834 | COG0409 | hypD | [O] | -2.4 | hydrogenase isoenzymes formation protein |
| CBO1058 | COG1331 |  | [O] | +2.2 | thymidylate kinase |
| CBO2439 | COG0606 |  | [O] | -2.8 | magnesium chelatase |
| CBO1069 | COG0492 |  | [O] | +2.7 | pyridine nucleotide-disulphide oxidoreductase |
| CBO3591 | COG1404 |  | [O] | -2.2 | alkaline serine protease |
| CBO0803 |  | ha-33 |  | +1.8 | toxin cluster gene ha-33 |
| CBO0802 |  | ha-17 |  | +1.6 | toxin cluster gene ha-17 |
| **Transcription [K]** | | | | | |
| CBO2961 | COG1420 | hrcA | [K] | +3.5 | heat-inducible transcription repressor |
| CBO0645 | COG1846 |  | [K] | +16.1 | MarR-family transcriptional regulator |
| CBO1012 | COG1846 |  | [K] | +7.7 | MarR-family transcriptional regulator |
| CBO1016 | COG1846 |  | [K] | -3.4 | MarR-family transcriptional regulator |
| CBO1017 | COG4978 |  | [KT] | -5.8 | MerR-family transcriptional regulator |
| CBO2304 | COG1695 |  | [K] | +5.8 | transcriptional regulator, PadR family |
| CBO2164 | COG2207 |  | [KS] | +3.9 | AraC family regulatory protein |
| CBO0786 | COG0745 |  | [KT] | +4.4 | two component response regulator |
| CBO3308 | COG3279 |  | [KT] | +3.9 | two-component response regulator |
| CBO0100 | COG1609 | ccpA | [K] | -7.3 | LacI-family transcriptional regulator |
| CBO1989 | COG2188 | treR | [K] | -3.6 | trehalose operon transcriptional repressor |
| CBO3497 | COG1595 | sigH | [K] | +3.5 | RNA polymerase sigma-H factor |
| CBO0043 | COG1595 | rpoE | [K] | +2.3 | RNA polymerase sigma factor |
| CBO2938 | COG0568 | rpoD | [K] | +4.2 | RNA polymerase sigma factor (sigma-43) |
| CBO3519 | COG0782 | greA | [K] | -5.0 | transcription elongation factor |
| CBO0624 | COG1396 |  | [K] | +2.3 | DNA-binding protein |
| CBO0847 | COG1396 |  | [K] | -2.5 | DNA-binding protein |
| CBO2530 | COG1327 |  | [K] | +3.2 | ATP-binding protein |
| CBO2755 | COG3711 |  | [K] | -2.8 | antiterminator |
| CBO0594 | COG3829 |  | [KT] | -3.3 | Sigma-54 interacting protein |
| **Translation, ribosomal structure and biogenesis [J]** | | | | | |
| CBO3164 | COG0525 | valS | [J] | -12.1 | valyl-tRNA synthetase |
| CBO3069 | COG0809 | queA | [J] | -9.2 | tRNA ribosyltransferase-isomerase |
| CBO1204 | COG0162 | tyrR | [J] | -5.6 | tyrosyl-tRNA synthetase |
| CBO3265 | COG0064 | gatB | [J] | -4.3 | glutamyl-tRNA amidotransferase subunit B |
| CBO3266 | COG0154 | gatA | [J] | -2.2 | glutamyl-tRNA amidotransferase subunit A |
| CBO3267 | COG0721 | gatC | [J] | -2.3 | glutamyl-tRNA amidotransferase subunit C |
| CBO3503 | COG0442 | proS2 | [J] | -4.3 | prolyl-tRNA synthetase |
| CBO1019 | COG0017 | aspS | [J] | -2.7 | aspartyl-tRNA synthetase |
| CBO3323 | COG0162 | tyrS | [J] | -4.7 | tyrosyl-tRNA synthetase |
| CBO0135 | COG0254 | rpmE | [J] | -2.3 | 50S ribosomal protein L31 |
| CBO3465 | COG0256 | rplR | [J] | -3.2 | 50S ribosomal protein L18 |
| CBO3462 | COG0200 | rplO | [J] | -3.8 | 50S ribosomal protein L15 |
| CBO3470 | COG0198 | rplX | [J] | -6.7 | 50S ribosomal protein L24 |
| CBO3472 | COG0186 | rpsQ | [J] | -2.1 | 30S ribosomal protein S17 |
| CBO3468 | COG0199 | rpsN | [J] | -2.5 | 30S ribosomal protein S14 |
| CBO3474 | COG0092 | rpsC | [J] | -4.6 | 30S ribosomal protein S3 |
| CBO3492 | COG0080 | rplK | [J] | -2.8 | 50S ribosomal protein L11 |
| CBO0400 | COG0465 | ftsH | [J] | -6.3 | cell division protein |
| CBO3521 | COG0042 |  | [J] | -3.2 | dihydrouridine synthase |
| **Amino acid transport and metabolism [E]** | | | | | |
| CBO1675 | COG0747 |  | [E] | +6.8 | oligopeptide-binding protein |
| CBO1676 | COG0601 |  | [EP] | +2.2 | oligopeptide transport system permease protein |
| CBO1677 | COG1173 |  | [EP] | +3.1 | oligopeptide transport system, permease protein |
| CBO1678 | COG0444 |  | [EP] | +2.7 | oligopeptide transport system, ATP-binding protein |
| CBO1860 | COG0697 | ecsC | [E] | +4.1 | membrane protein |
| CBO3174 | COG1305 |  | [E] | +2.3 | integral membrane protein |
| CBO1167 | COG0697 |  | [EG] | +2.6 | membrane protein |
| CBO1802 | COG1167 |  | [EK] | +4.0 | aminotransferase |
| CBO1400 | COG1168 |  | [E] | -2.6 | aminotransferase |
| CBO0344 | COG0006 |  | [E] | -3.2 | probable metallopeptidase |
| CBO1539 | COG3104 |  | [E] | -2.5 | probable transporter |
| CBO2233 | COG2008 |  | [E] | +3.5 | threonine aldolase |
| **Cell wall/membrane/envelope biogenesis [M]** | | | | | |
| CBO1424 | COG0860 |  | [M] | -3.0 | N-acetylmuramoyl-L-alanine amidase |
| CBO2101 | COG1087 |  | [M] | -4.2 | UDP-glucose epimerase |
| CBO1454 | COG0275 | mraW | [M] | -2.1 | S-adenosyl-methyltransferase |
| CBO3562 | COG0796 | dga | [M] | +3.1 | glutamate racemase |
| CBO1660 | COG0787 | alr | [M] | +3.6 | alanine racemase |
| CBO0380 | COG2247 |  | [M] | +2.6 | cell surface protein |
| CBO3597 | COG0764 - | fabZ | [M] | +8.3 | (3R)-hydroxymyristoyl-ACP dehydratase |
| CBO3599 | COG0304 | fabF | [M] | +2.4 | 3-oxoacyl-ACP synthase II |
| CBO3600 | COG1028 | fabG | [M] | +2.9 | 3-oxoacyl-ACP reductase |
| CBO3548 | COG0773 | murC | [M] | -2.3 | UDP-N-acetylmuramate--L-alanine ligase |
| CBO3606 | COG0739 |  | [M] | +2.5 | exported peptidase |
| **Hypothetical proteins** | | | | | |
| CBO3520 |  |  |  | -2.3 | hypothetical protein |
| CBO3522 |  |  |  | -4.1 | hypothetical protein |
| CBO0008 |  |  |  | +2.3 | hypothetical protein |
| CBO2185 |  |  |  | +5.3 | hypothetical protein |
| CBO0443 |  |  |  | +2.0 | hypothetical protein |
| CBO2640 |  |  |  | -6.3 | hypothetical protein |
| CBO2179 |  |  |  | -2.5 | hypothetical protein |
| CBO1858 |  |  |  | -3.8 | hypothetical protein |
| CBO2096 |  |  |  | +5.8 | hypothetical protein |
| CBO3353 |  |  |  | +7.6 | hypothetical protein |
| CBO1237 |  |  |  | +2.1 | hypothetical protein |
| CBO0578 |  |  |  | -3.0 | hypothetical protein |
| CBO2697 |  |  |  | -2.4 | hypothetical protein |
| CBO2693 |  |  |  | -2.3 | hypothetical protein |
| CBO0649 |  |  |  | +3.2 | hypothetical protein |
| CBO0922 |  |  |  | +3.1 | hypothetical protein |
| CBO3007 |  |  |  | -4.0 | hypothetical protein |
| CBO2314 |  |  |  | -2.1 | hypothetical protein |
| CBO3038 |  |  |  | +2.0 | hypothetical protein |
| CBO0034 |  |  |  | -2.6 | hypothetical protein |
| CBO0569 |  |  |  | -2.4 | hypothetical protein |
| CBO1074 |  |  |  | +3.2 | hypothetical protein |
| **Function unknown [S]** | | | | | |
| CBO1070 | COG3862 |  | [S] | +6.0 | hypothetical protein |
| CBO3129 | COG3027 |  | [S] | -3.6 | hypothetical protein |
| CBO0014 | COG3862 |  | [S] | -4.2 | hypothetical protein |
| CBO3570 | COG1576 |  | [S] | +2.6 | hypothetical protein |
| CBO3588 | COG4508 |  | [S] | +3.0 | hypothetical protein |
| CBO2935 | COG0327 |  | [S] | +4.1 | hypothetical protein |
| CBO3194 | COG3581 |  | [S] | -2.1 | hypothetical protein |
| CBO1534 | COG5441 |  | [S] | -2.7 | hypothetical protein |
| CBO1128 | COG4198 |  | [S] | +2.6 | hypothetical protein |
| CBO0346 | COG4378 |  | [S] | +2.5 | hypothetical protein |
| CBO2339 | COG5280 |  | [S] | -2.9 | hypothetical protein |
| CBO0723 | COG0693 |  | [R] | +2.6 | hypothetical protein |
| CBO2075 | COG2108 |  | [R] | +2.7 | hypothetical protein |
| CBO3175 | COG2137 |  | [R] | -2.6 | hypothetical protein |
| CBO3617 | COG2509 |  | [R] | -2.6 | hypothetical protein |
| **General function prediction only [R]** | | | | | |
| CBO1023 | COG1011 |  | [R] | +2.9 | hydrolase |
| CBO1449 | COG2234 |  | [R] | +4.6 | membrane protein |
| CBO0176 | COG2234 |  | [R] | -3.2 | aminopeptidase |
| CBO0670 | COG2984 |  | [R] | -4.2 | lipoprotein |
| CBO3350 | COG1277 |  | [R] | -2.8 | lipoprotein |
| CBO3554 | COG1242 |  | [R] | -3.2 | radical SAM superfamily protein |
| CBO2901 | COG2252 |  | [R] | +2.8 | purine transporter |
| CBO0849 | COG0596 |  | [R] | +2.0 | non-heme chloroperoxidase |
| CBO0530 | COG0663 |  | [R] | +3.8 | transferase |
| CBO3176 | COG0612 |  | [R] | -2.8 | zinc-binding protease |
| CBO1722 | COG1783 |  | [R] | -2.9 | terminase large subunit |
| CBO2137 | COG2234 |  | [R] | +4.7 | membrane protein |
| **Cell motility [N]** | | | | | |
| CBO2645 | COG1298 | flhA | [NU] | -7.9 | flagellar biosynthesis protein |
| CBO2652 | COG1291 | motA | [N] | -5.2 | Chemotaxis MotA protein |
| CBO2651 | COG1360 | motB | [N] | -2.2 | Chemotaxis MotB protein |
| CBO2744 | COG1868 | fliM | [N] | -3.0 | flagellar motor switch protein FliM |
| CBO2658 | COG2882 | fliJ | [NUO] | -2.1 | flagellar protein |
| CBO2734 | COG1516 | fliS | [NUO] | -3.5 | flagellar protein FliS |
| **Energy production and conversion [C]** | | | | | |
| CBO1068 | COG0579 | glpA | [RC] | +3.1 | anaerobic glycerol-3-phosphate dehydrogenase subunit A |
| CBO1840 | COG0374 | hupL | [C] | -2.2 | hydrogenase large subunit |
| CBO3183 | COG1882 |  | [C] | -3.9 | pyruvate formatelyase |
| CBO3184 | COG1180 |  | [C] | -3.6 | pyruvate formate-lyase 2 activating enzyme |
| CBO3577 | COG0778 |  | [C] | -5.2 | nitroreductase |
| CBO0155 | COG0224 | atpG | [C] | -2.6 | ATP synthase subunit gamma |
| CBO0207 | COG1600 |  | [C] | -2.5 | iron-sulfur cluster-binding protein |
| CBO2623 | COG1394 | ntpD | [C] | +2.3 | v-type sodium ATP synthase subunit D |
| CBO3283 | COG1052 | ldhA1 | [CH] | +4.0 | D-lactate dehydrogenase |
| CBO1490 | COG1757 |  | [C] | +2.3 | Na+/H+ antiporter |
| CBO3268 | COG1301 |  | [C] | -2.9 | integral membrane transport protein |
| **Signal transduction mechanisms [T]** | | | | | |
| CBO0787 | COG0642 |  | [T] | +6.5 | two-component sensor kinase |
| CBO3309 | COG0642 |  | [T] | +3.6 | two-component sensor kinase |
| CBO1213 | COG2199 |  | [T] | -3.5 | signaling protein |
| CBO1120 | COG0642 |  | [T] | +4.3 | two-component sensor kinase |
| CBO0910 | COG1974 |  | [T] | -2.6 | serine peptidase |
| **Cell cycle control, cell division, chromosome partitioning [D]** | | | | | |
| CBO2535 | COG0206 | ftsZ | [D] | -4.7 | cell division protein FtsZ |
| CBO0088 | COG0489 |  | [D] | -2.0 | ATP-binding protein |
| CBO2809 | COG3584 |  | [DS] | -3.1 | exported protein |
| **Lipid transport and metabolism [I]** | | | | | |
| CBO3598 | COG0511 | accB | [I] | +3.2 | biotin carboxyl carrier protein of acetyl-coa carboxylase |
| CBO2187 | COG0657 |  | [I] | -2.6 | exported protein |
| CBO3195 | COG1924 |  | [I] | -2.4 | activator of 2-hydroxyacyl-CoA-hydratase |
| **Coenzyme transport and metabolism [H]** | | | | | |
| CBO2911 | COG0422 | thiC | [H] | -4.3 | thiamine biosynthesis protein |
| CBO0936 | COG1903 | cbiD | [H] | -2.0 | cobalamin biosynthesis protein |
| CBO1339 | COG1120 |  | [HP] | -3.1 | iron ABC transporter, ATP-binding protein |
| CBO0377 | COG1477 |  | [H] | +2.5 | lipoprotein |
| CBO2501 | COG1564 |  | [H] | +2.3 | thiamine pyrophosphokinase |
| CBO2601 | COG1057 |  | [H] | +4.6 | transferase |
| CBO1933 | COG0476 | chlN | [H] | +2.8 | molybdopterin biosynthesis protein |
| CBO0919 | COG1648 |  | [H] | -2.5 | precorrin oxidase |
| Nucleotide transport and metabolism [F] | | | | | |
| CBO3295 | COG0518 | guaA | [F] | +3.5 | GMP synthase [glutamine-hydrolyzing] |
| CBO2876 | COG0034 | purF | [F] | +2.4 | amidophosphoribosyltransferase precursor |
| CBO2894 | COG1618 |  | [F] | +2.0 | nucleoside triphosphatase |
| CBO0132 | COG0504 | pyrG | [F] | -4.7 | CTP synthase |
| **Carbohydrate transport and metabolism [G]** | | | | | |
| CBO1071 | COG2271 | glpT | [G] | +4.3 | glycerol-3-phosphate transporter |
| CBO2398 | COG1925 | ptsH | [G] | +2.4 | phosphocarrier protein HPr |
| CBO3438 | COG1080 | ptsI | [G] | +2.6 | phosphoenolpyruvate-protein phosphotransferase |
| CBO2688 | COG4464 |  | [GM] | -2.2 | protein-tyrosine phosphatase |
| CBO1602 | COG1940 | glcK | [GK] | -2.5 | glucokinase |
| **Intracellular trafficking, secretion, and vesicular transport [U]** | | | | | |
| CBO3569 | COG0653 |  | [U] | +2.7 | preprotein translocase |
| **Inorganic ion transport and metabolism [P]** | | | | | |
| CBO1086 | COG1464 | metQ1 | [P] | -2.3 | D-methionine-binding lipoprotein |
| CBO0841 | COG4300 |  | [P] | -2.6 | efflux protein |
| **Secondary metabolites biosynthesis, transport and catabolism [Q]** | | | | | |
| CBO1169 | COG2313 |  | [Q] | +4.3 | indigoidine synthase A family protein |
| **Replication, recombination and repair [L]** | | | | | |
| CBO0073 | COG1573 |  | [L] | +2.0 | uracil-DNA glycosylase |
